# Estimating the joint effect of diabetes and subsequent depressive symptoms on mortality among older latinos

**DOI:** 10.1016/j.annepidem.2021.09.007

**Published:** 2021-09-24

**Authors:** Kosuke Inoue, Elizabeth Rose Mayeda, Roch Nianogo, Kimberly Paul, Yu Yu, Mary Haan, Beate Ritz

**Affiliations:** a Department of Epidemiology, UCLA Fielding School of Public Health, Los Angeles, CA; b Department of Social Epidemiology, Graduate School of Medicine, Kyoto University, Japan; c Department of Epidemiology & Biostatistics, School of Medicine, UCSF, San Francisco, CA; d Department of Environmental Health Sciences, UCLA Fielding School of Public Health, Los Angeles, CA; e Department of Neurology, UCLA David Geffen School of Medicine, Los Angeles, CA

**Keywords:** Longitudinal study, Diabetes, Depression, Cardiovascular mortality, Marginal structural model, Latinos

## Abstract

**Purpose:**

Diabetes and depression are risk factors for cardiovascular disease, but the evidence about their interaction effect on long-term health outcomes among Latinos is lacking. We aimed to investigate the joint association of diabetes and subsequent depressive symptoms with mortality among older Latinos, an understudied racial/ethnic group with high prevalence of diabetes.

**Methods:**

This study included 1,495 adults from the Sacramento Area Latino Study on Aging. We employed Cox proportional-hazards models to estimate the adjusted hazard ratios [aHRs] for cardiovascular and all-cause mortality according to diabetes status at enrollment and depressive symptoms a year after the enrollment. We used marginal structural models to adjust for time-varying confounders.

**Results:**

The mean age (standard deviation) of participants was 70 (6.6) years. Over follow-up (median 7.7 years), diabetes and depressive symptoms were individually associated with increased risk of cardiovascular mortality (diabetes, aHR[95% CI]=2.13[1.60–2.84]; depressive symptoms, aHR[95% CI]=1.62[1.09–2.39]) and all-cause mortality (diabetes, aHR[95% CI]=1.92[1.53–2.41]; depressive symptoms, aHR[95% CI]=1.41[1.02–1.94]). After adjusting for time-varying confounders, we found a multiplicative interaction between diabetes and subsequent depressive symptoms for cardiovascular mortality (aHR[95% CI]=2.94[1.07–8.39]), but not all-cause mortality (aHR[95% CI]=1.80[0.81–4.35]).

**Conclusions:**

Using a longitudinal cohort of community-dwelling older Latinos, we found that diabetes and subsequent depressive symptoms were jointly associated with increased risk of cardiovascular mortality.

## Introduction

The public health burden of cardiovascular disease (CVD) and its risk factors in US Latinos have received substantial attention. In 2014, the American Heart Association published a statement calling for the development of culturally tailored and targeted approaches to improve cardiovascular health and reduce CVD events among this population [1]. Diabetes is one of the major causes of CVD, affecting 14.3 million, or more than one in four US adults older than 64 years in 2018 [2]. Importantly, the prevalence of diabetes varies by race/ethnicity, with nearly double the prevalence of diabetes among Latinos compared with non-Latino whites [2,3]. Further-more, diabetes is the fifth leading cause of death with 145.4 cases per 100,000 population among Latinos ages 65 and older, while it is the seventh leading cause of death with 103.5 cases per 100,000 population among older non-Latino whites [2,4]. Racial/ethnic disparities in access to care and differences in adherence to treatment [5–7] may contribute to the higher prevalence of diabetes and its complications among Latinos in the US. However, to design effective policy and clinical interventions that reduce racial health disparities, greater insight into factors that act as mediators and/or effect measure modifiers on the causal pathway from diabetes to CVD and death—particularly among older Latinos, a large but understudied racial/ethnic group in the US—are needed.

Depression is a well-known factor closely associated with diabetes and CVD [8–11]. The prevalence of depression in people with type 2 diabetes is almost double that of people without diabetes [12]. A recent meta-analysis showed that depression was associated with increased risk of CVD events among people with type 2 diabetes, and the association was robust to potential uncontrolled confounding such as employment status [13]. However, most previous studies based on conventional regression analyses have not provided sufficient insight into (i) the temporal ordering between diabetes and depression and (ii) time-varying confounders between these conditions and CVD (e.g., hypertension, obesity, and dyslipidemia). Given that depression can be both a cause or effect of diabetes [8,9,14] and they are closely interrelated with other metabolic disorders, further evidence from longitudinal studies with clear time-ordering of these diseases is warranted to evaluate the potential synergistic impact of diabetes and depression on CVD and death.

Therefore, using a longitudinal cohort of community-dwelling older Latinos, along with marginal structural models (MSMs), we investigated the joint association of diabetes and subsequent depressive symptoms (after a diagnosis of diabetes) with cardiovascular and all-cause mortality. Fitting MSMs allows us to estimate the joint effect of two exposures at different time points (i.e., diabetes at enrollment and subsequent depressive symptoms a year after the enrollment) on outcomes (i.e., cardiovascular and all-cause mortality) in the presence of time-varying covariates that are simultaneously confounders and intermediate variables [15].

## Methods

### Study design and patients

**Fig. 1.**
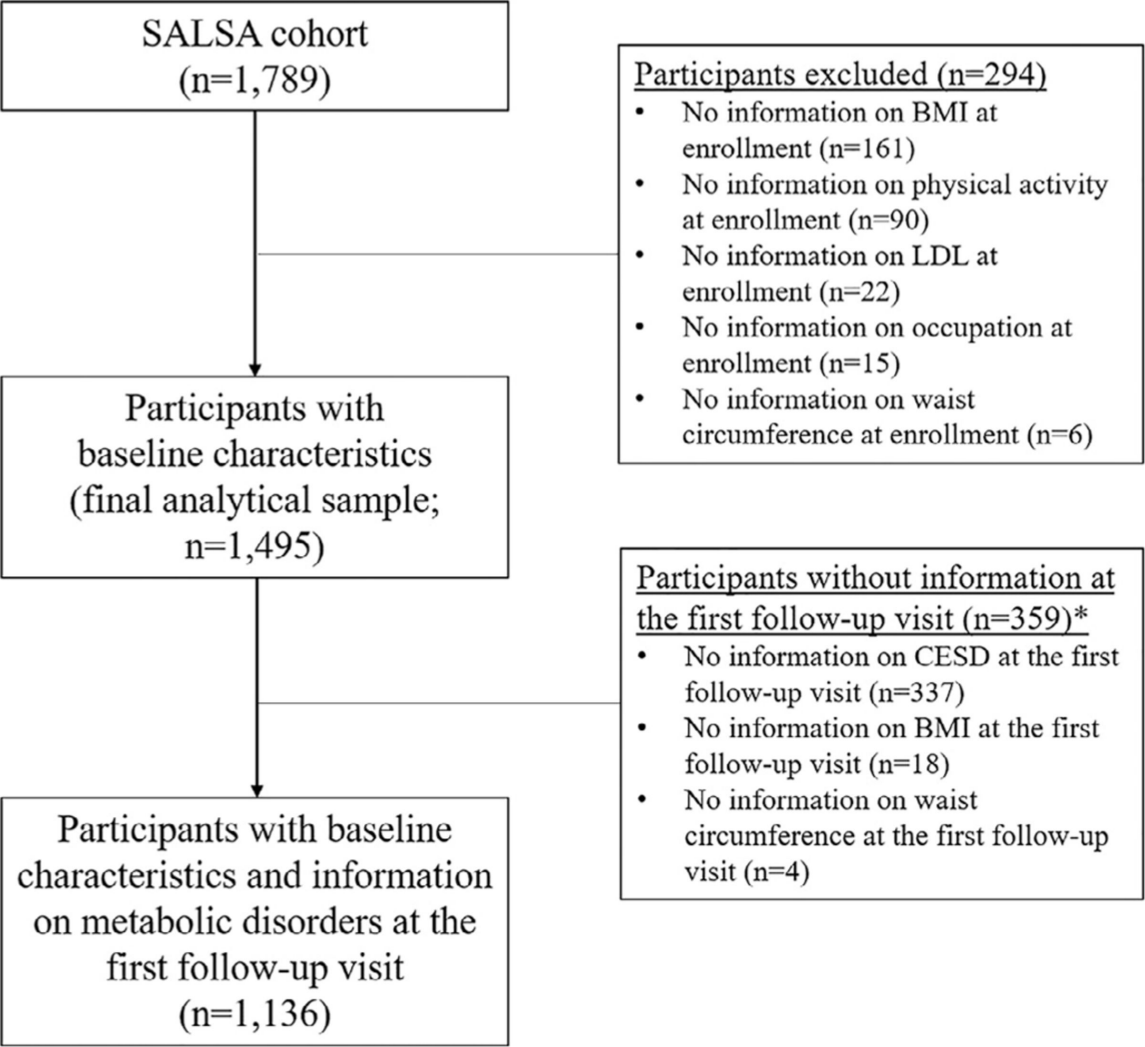
The flow of study population, Sacramento Area Latino Study on Aging (SALSA). ^∗^Inverse-probability-censoring weights were applied to adjust for the right censoring at the follow-up visit due to loss to follow-up (*n* = 359).

All study participants were enrolled in the Sacramento Area Latino Study on Aging (SALSA), a population-based prospective cohort of Latinos aged 60 years and older. Eligibility criteria included (i) being 60 years of age or older at the time of enrollment in 1998–1999, (ii) residing in a six-county area in the Sacramento Valley region (Sacramento, Yolo, Sutter, Solano, San Joaquin, and Placer counties), and (iii) self-identification as Latino, Mexican, Central American, or Mexican American. Follow-up interviews and exams were conducted at their homes every 12–15 months for up to seven study visits by the end of 2007. More details about sampling and study procedures can be found elsewhere [16]. Among 1789 participants enrolled in SALSA, a total of 1495 participants (84%) with complete data on the covariates at enrollment (mentioned below) were included in this study (Fig. 1). The primary language was English for 665 (44%) participants and Spanish for 830 (56%) participants.

### Measurement of variables

#### Diabetes

Diabetes was classified based on fasting glucose level ≥126 mg/dL (≥7 mmol/L), antidiabetic medication use, or self-report of a physician diagnosis at enrollment using the same definition in previous studies [17,18]. Fasting glucose was measured with the Cobas Mira Chemistry Analyzer (Roche Diagnostics Corporation, Indianapolis, IN). Diabetic medications were assessed by a medicine cabinet inventory of prescription medicines. Given the age of this cohort, we assumed most (if not all) cases were type 2 rather than type 1 diabetes [17,18].

#### Depressive symptoms

The Center for Epidemiological Studies-Depression (CESD) scale, one of the most widely used measurement tools for depressive symptoms in geriatric populations [19] and older Latinos [20], was administered to all SALSA participants [21]. The CESD consists of 20 four-point (0, 1, 2, 3) Likert-type questions (total score: 0 – 60). We considered a participant to have elevated depressive symptoms when CESD was ≥16 (standard cutoff score) or they used antidepressants [22]. Antidepressant use was assessed by a medicine cabinet inventory of prescription medicines. Both CESD and antidepressant use were assessed at enrollment and the first follow-up visit (around a year after the enrollment)—hereafter referred to as “at follow-up.”

#### Cardiovascular and all-cause mortality

The primary outcome was all-cause mortality, and the secondary outcome was cardiovascular mortality (including stroke). We ascertained mortality data through December 2007, using online obituary surveillance, review of the Social Security Death Index and the National Death Index, review of vital statistics data files from California, and interviews with family members. If a participant was not identified as dead, they were censored at the date of the last contact. Cardiovascular mortality was defined based on the International Classification of Diseases, Tenth version (ICD–10); ischemic heart diseases (I20–I25), heart failure (I50), and stroke (I63–64). If a death certificate was not located, the death was coded as all-cause mortality with unspecified cause [23].

#### Other covariates

At baseline, participants provided sociodemographic information including age, sex, country of birth, years of education, marital status, and type of lifetime occupation (non-manual, manual, and others). We also included health behaviors related to diabetes, depression, and mortality, including smoking status, any alcohol use, and physical activity levels (METS-hour/week). Cumulative METS-hour/week was calculated by summing the self-reported average number of hours spent on nine non-occupational activities with moderate-vigorous intensity as previously described [18]. Waist circumference (inches) was measured at the level of maximum indentation over the abdomen. We calculated body mass index (BMI, kg/m^2^) based on measured height with a tape measure and weight on a Tanita scale. Hypertension was based on measured systolic blood pressure (≥140 mmHg), diastolic blood pressure (≥90 mmHg), self-report of physician diagnosis, and/or anti-hypertensives use. A previous history of CVD (including stroke) was also self-reported. Low-density lipoprotein (LDL) cholesterol was measured from morning fasting serum samples using the LDL Direct Liquid Select (Equal Diagnostics, Exton, Pennsylvania). Statin prescription was assessed using the same approach with antidiabetic medication and antidepressants. BMI, waist circumference, hypertension, previous history of CVD, and statin prescription were also assessed at the follow-up visit.

#### Statistical analyses

**Fig. 2.**
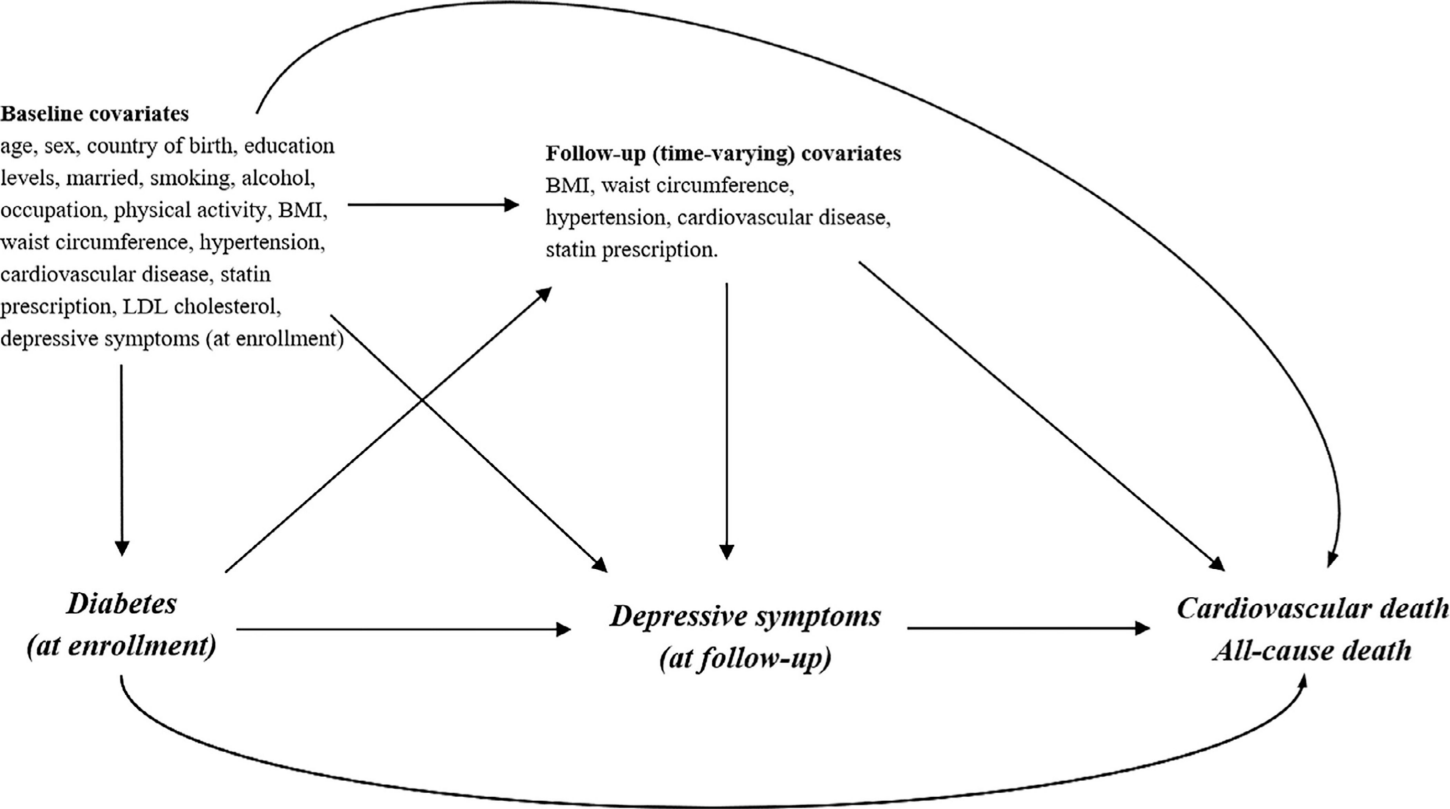
Diagram illustrating proposed causal structure between diabetes status at enrollment, depressive symptoms at follow-up, and mortality, including time-varying metabolic disorders at enrollment and follow-up.

We described the distribution of CESD at the follow-up visit according to diabetes status at enrollment. We subsequently employed Cox proportional hazard models to estimate hazard ratios (HR) of cardiovascular and all-cause mortality according to diabetes status and according to having elevated depressive symptoms at the follow-up visit. We selected potential confounders at enrollment and the follow-up visit *a priori* considering factors that may affect each outcome (i.e., cardiovascular or all-cause mortality) and might also be associated with diabetes at enrollment and elevated depressive symptoms at the follow-up visit (Fig. 2).

Utilizing marginal structural Cox models with inverse-probability-of-treatment weights (IPTW) [24], we investigated the association of diabetes at enrollment with cardiovascular and all-cause mortality accounting for the intermediary role of subsequent depressive symptoms at the follow-up visit (i.e., after the assessment of diabetes status). Given the potential bias due to loss to follow-up, we also employed the inverse-probability-of-censoring weights (IPCW) assuming that diabetes status and covariates at enrollment in Fig. 2 might have affected the censoring at the follow-up visit. The final weights for each participant were created by multiplying the IPTW and the IPCW (Supplementary Text). Multiplicative interaction was estimated by inserting an interaction term between diabetes and subsequent depressive symptoms in the regression models, and additive interaction was estimated using the relative excess risk due to interaction (RERI). Robust 95% confidence intervals (CIs) were estimated by repeating these analyses on 1000 bootstrapped samples.

We conducted the following two additional analyses. First, to compare the results from models with and without adjusting for time-varying metabolic disorders, we analyzed the data using diabetes status and elevated depressive symptoms at enrollment (i.e., without adjusting for time-varying metabolic disorders). Second, to minimize the possibility of reverse causation, we restricted analyses to participants without depressive symptoms at enrollment. Statistical analyses were conducted using R version 4.0.2.

## Results

At enrollment, the average age (standard deviation) of participants was 70 (6.6) years, and 41% were male (Table 1). Among 488 participants with diabetes, 327 (67%) received antidiabetic therapies and 143 (29%) had fasting glucose levels <126 mg/dl at the baseline assessment. Individuals with diabetes at enrollment generally were more likely to be born in the US, have higher BMIs and waist circumferences, have a higher prevalence of hypertension and CVD, and have a higher prevalence of statin and antidepressant use, while individuals free of diabetes at enrollment were more likely to be current smokers, report consuming alcohol, and be physically active. Among 1495 participants included in our study, 1136 participants (76%) completed the information on depressive symptoms and metabolic disorders at the follow-up visit with the median duration of 1.08 (interquartile range, 0.97–1.25) years from enrollment. At the follow-up visit, we found similar patterns of characteristics by diabetes; i.e., individuals with diabetes at enrollment tended to have higher BMI and waist circumference, have a higher prevalence of hypertension and CVD, and higher prevalence of statin and antidepressant use at the follow-up visit compared with individuals free of diabetes at enrollment.

The CESD at the follow-up visit showed a right-skewed distribution, with a higher prevalence of low CESD among individuals free of diabetes than those with diabetes at enrollment (Supplementary Figure 1). The percentage of participants with elevated depressive symptoms at the follow-up visit was 25% (286/1136) among the total study population, 30% (107/356) among those with diabetes at enrollment, and 23% (179/780) among those without diabetes at enrollment (Supplementary Table 1). Among 286 participants with elevated depressive symptoms, 104 (36%) used antidepressants.

The median duration of mortality follow-up was 7.7 (interquartile range, 5.0–8.2) years. During these periods, 218 (15%) participants died from cardiovascular disease, and 341 (23%) died from all causes in the total study population. Individuals reporting diabetes at enrollment were at increased risk of cardiovascular mortality (HR, 2.13; 95% CI, 1.60 – 2.84) and all-cause mortality (HR, 1.92; 95% CI, 1.53 – 2.41) after adjusting for potential confounders at enrollment (Table 2). Individuals with elevated depressive symptoms at the follow-up visit were also at increased risk of cardiovascular mortality (HR, 1.62; 95% CI, 1.09 to 2.39) and all-cause mortality (HR, 1.41; 95% CI, 1.02 – 1.94) after adjusting for potential confounders at enrollment and the follow-up visit.

In MSMs adjusting for potential confounders (including depressive symptoms at enrollment), individuals reporting diabetes at enrollment without depressive symptoms at the follow-up visit were at increased risk of cardiovascular (HR, 1.82; 95% CI, 1.12 –3.02) and all-cause mortality (HR, 2.10; 95% CI, 1.36 – 3.30) (Table 3). Individuals reporting diabetes at enrollment with elevated depressive symptoms at the follow-up visit were at even greater risk of cardiovascular (HR, 5.78; 95% CI, 3.02 – 11.97) and all-cause mortality (HR, 4.32; 95% CI, 2.41 – 7.31). Multiplicative interaction between diabetes and subsequent depressive symptoms was found for cardiovascular mortality (HR, 2.94; 95% CI, 1.07 –8.39) but not for all-cause mortality (HR, 1.80; 95% CI, 0.81 –4.35). Additive interactions were found for both cardiovascular mortality (RERI, 3.79; 95% CI, 1.05 – 9.81) and all-cause mortality (RERI, 2.02; 95% CI, 0.01 – 5.08).

Results remained qualitatively consistent when evaluating diabetes and elevated depressive symptoms at the same time point, but we did not find interactions between diabetes and elevated depressive symptoms for cardiovascular and all-cause mortality (Supplementary Table 2); in fact, the estimated joint effects of diabetes and elevated depressive symptoms were underestimated compared to the analysis in which we evaluated diabetes and subsequent depressive symptoms adjusting for time-varying confounders. The results did not qualitatively change when we restricted the analysis to participants without depressive symptoms at enrollment although it did not have sufficient statistical power due to small sample size in each stratum (Supplementary Table 3).

## Discussion

In this longitudinal population-based study of older Latinos, adjusting for time-varying metabolic disorders, we found a joint association of diabetes at baseline and subsequent elevated depressive symptoms after one year of follow-up with cardiovascular mortality. Although this observational study is not sufficient to establish causality, our findings advance our current state of knowledge and provide novel insight into the potential impact of adverse mental health after a diagnosis of diabetes on cardiovascular health outcomes.

Racial/ethnic disparities in CVD management have been one of the major public health issues in the US. Some previous studies have shown that Latinos had a lower risk of cardiovascular mortality despite their higher prevalence of CVD risk factors compared with non-Latino whites [25], which is sometimes discussed in the context of the “Hispanic paradox” [26,27]. Diabetes has been shown to be more weakly associated with CVD but more strongly with end-stage renal disease and mortality among Latinos compared to non-Latino whites [6,7]. Moreover, in our study, the prevalence of antidiabetic medication use among those with diabetes was 67%, which was lower than the 75% reported for adults aged ≥65 years with diabetes in the US general population [28]. Given such apparent inadequate care and paradoxes for Latinos, it is imperative to better understand the forces that drive diabetes, depression, and CVD in this large racial/ethnic group living in the US with a high prevalence of diabetes.

Our findings corroborate a previously reported joint association for diabetes and depression with cardiovascular and all-cause mortality [13], and extend the evidence to older Latinos. A large case-control study revealed that after lipids and smoking, psychosocial stress, including depression, was the third factor contributing greatly to the attributable risk of acute myocardial infarction (approximately 30%) [29]. In addition, previous cohort studies showed that comorbid depression was associated with an increased risk of CVD and all-cause mortality among US veterans with diabetes [30,31]. Moreover, studies have consistently reported that people affected by both diabetes and depression showed the strongest cardiovascular and all-cause mortality risks compared to people without diabetes and depression [31–35]. The findings were also true for older Latinos enrolled in another cohort study (the Hispanic Established Population for the Epidemiologic Study of the Elderly survey) following its participants from 1995 through 2001 [36]. However, these studies did not address the temporal order between diabetes and depression and failed to control for time-varying confounders such as metabolic disorder status. The smaller joint effects we estimated in our analysis evaluating diabetes and elevated depressive symptoms at the same time point compared to our analysis that evaluated diabetes and subsequent depressive symptoms suggest that prior findings might have underestimated the potential harmfulness of depressive symptoms among people with diabetes due to ill-defined temporality and insufficient control for confounding. In this context, our study contributes uniquely to the literature as we addressed the temporal ordering of diabetes and elevated depressive symptoms and took time-varying metabolic disorders into account.

Several mechanisms underlying the relationship between depressive symptoms and CVD have been established [37]. Depressive symptoms are known to activate the hypothalamic-pituitary-adrenal axis by releasing corticotropin-releasing factors from the hypothalamus subsequently increasing corticosteroids, which induce atherosclerosis, hypertension, and dyslipidemia [8,37]. They also decrease parasympathetic nervous system responses, which lower heart-rate variability leading to dysrhythmia [38]. Other potential biological mechanisms that mediate the effect of depressive symptoms on CVD include inflammatory activity, endothelial dysfunction, and platelet dysfunction [39–42]. These dysfunctions may exacerbate the consequences of diabetes, metabolic syndrome, and insulin resistance, increasing the risk of having a CVD event [43]. Moreover, behavioral mechanisms play an important role in this relationship between depressive symptoms and CVD because depressive symptoms decrease adherence to medication and a healthy lifestyle such as exercise, healthy diets, and smoking cessation, all of which are protective factors for CVD [37,44,45]. These mechanisms are also strongly related to diabetes and its complications [8], and therefore, may contribute to the synergistic effect of diabetes and depression on cardiovascular mortality. Identification and quantification of biological and behavioral mechanisms from diabetes to depression leading to death should be the subject of future studies.

A major strength of the present study is its population-based longitudinal design and follow-up of older Latinos for about a decade that allowed us to investigate the incidence of cardiovascular and all-cause mortality in an understudied racial/ethnic group. Moreover, this resource helped us clarify the temporal relationship between diabetes and elevated depressive symptoms while at the same time adjusting for time-varying metabolic disorders employing IPTW. We further utilized IPCW to account for potential bias due to loss to follow-up. However, our study has several limitations. First, our findings may not be generalizable to institutionalized older Latinos or those living outside the Sacramento Area. Second, cohort participants had to survive to at least 60 years of age to be enrolled, and therefore, our results might have been biased due to the exclusion of people who died before age 60 years. Third, because of the small number of events in each stratum, our results showed wide 95% CI suggesting the need for larger studies to validate our findings. Fourth, although we adjusted for an extensive set of potential confounders, there is a potential for uncontrolled or residual confounding due to the nature of the observational design. Fifth, because we classified diabetes based on self-report, medication, and fasting glucose levels, we cannot rule out the possibility of misclassification of this exposure. We did not have information about types of diabetes (i.e., type 1, type 2, or others), diabetes severity, duration of diabetes, and whether depressive symptoms and mortality were directly related to diabetes. There is also a possibility of misclassification of CVD mortality because the cause of mortality was determined by ICD-10 code. Lastly, given that 294 participants were excluded due to missing data on baseline covariates, selection bias needs to be acknowledged, particularly if missing was not at random given covariates included in our model. In addition, across a total of 1495 participants included in our study, 24% of participants did not return for the follow-up visits. We applied IPCW to minimize the bias due to loss to follow-up, but selection bias could still be present if there were unmeasured variables related to death/drop out and there was differential death/drop out according to diabetes status and/or depressive symptoms.

In conclusion, using the longitudinal cohort with clear temporal ordering between diabetes and elevated depressive symptoms, along with employing methods to adjust for time-varying metabolic disorders, we found that diabetes and subsequent depressive symptoms were jointly associated with an increased risk of cardiovascular mortality among community-dwelling older Latinos. Future studies are needed to illuminate whether and what kind of clinical interventions to reduce depressive symptoms after diabetes are beneficial to promote cardiovascular health within the older Latino community.

## Supplementary Material

Supplementary materials

## Figures and Tables

**Table 1:** Characteristics of SALSA participants according to diabetes status at enrollment^*^.

Variable	Diabetes at enrollment (*n* = 488)	Free of diabetes at enrollment (*n* = 1007)
A) Baseline information		

Age (years old)	69.9 ± 6.5	70.2 ± 6.7
Male, N (%)	213 (43.7)	405 (40.2)
US born, N (%)	280 (57.4)	472 (46.9)
Education years, N (%)		
0	68 (13.9)	117 (11.6)
1–8	220 (45.1)	478 (47.5)
9–12	117 (24.0)	234 (23.2)
≥13	83 (17.0)	178 (17.7)
Married, N (%)	295 (60.5)	576 (57.2)
Type of occupation, N (%)		
Non-manual	109 (22.3)	220 (21.9)
Manual	287 (58.8)	596 (59.2)
Other	92 (18.9)	191 (19.0)
Smoking, N (%)		
Current	45 (9.2)	126 (12.5)
Former	229 (46.9)	412 (40.9)
Never	214 (43.9)	469 (46.6)
Alcohol consumption, N (%)	203 (41.6)	622 (61.8)
Physical Activity (METs per week)	64.3 ± 72.5	75.2 ± 74.4
BMI (kg/m^2^)	31.1 ± 6.3	29.2 ± 5.6
Waist circumference (inches)	39.9 ± 4.8	37.4 ± 5.2
Hypertension, N (%)	400 (82.0)	637 (63.3)
Cardiovascular diseases, N (%)	234 (48.0)	308 (30.6)
LDL Cholesterol (mg/dL)	117 ± 35	126 ± 34
Statin use, N (%)	54 (11.1)	82 (8.1)
CESD scale	10.7 ± 10.7	9.5 ± 10.4
Anti-depressant use, N(%)	55 (11.3)	69 (6.9)

B) Follow-up information (at the first follow-up visit)	Diabetes at enrollment (*n* = 356)	Free of diabetes at enrollment (*n* = 780)

Years from enrollment to the first follow-up	1.12 ± 0.21	1.13 ± 0.21
BMI (kg/m^2^)	31.0 ± 6.5	29.2 ± 5.8
Waist circumference (inches)	39.6 ± 5.2	37.6 ± 5.2
Hypertension, N (%)	310 (87.1)	574 (73.6)
Cardiovascular diseases, N (%)	198 (55.6)	270 (34.6)
Statin use, N (%)	62 (17.4)	103 (13.2)
CESD scale	9.7 ± 10.4	7.9 ± 9.6
Anti-depressant use, N(%)	41 (11.5)	63 (8.1)

SALSA = Sacramento Area Latino Study on Aging; METs = metabolic equivalent for task; BMI= body mass index; LDL= low-density lipoprotein; CESD= the Center for Epidemiological Studies-Depression.

*a Data are presented as count (percentage) or mean ± standard deviation otherwise indicated.

**Table 2:** Associations of (A) diabetes and (B) elevated depressive symptoms at the follow-up visit with cardiovascular mortality and all-cause mortality

Outcomes	Cardiovascular mortality		All-cause mortality	
		
Exposures	Number of events	Adjusted HR (95% CI)	Number of events	Adjusted HR (95% CI)
A) Diabetes at enrollment^*^				
No	102/1007	Ref	178/1007	Ref
Yes	116/488	2.13 (1.60 – 2.84)	163/488	1.92 (**1.53** – 2.41)
B) Elevated depressive symptoms at the follow-up visit^†^				
No	93/850	Ref	150/850	Ref
Yes	55/286	1.62 (1.09 – 2.39)	81/286	1.41 (1.02 – 1.94)

HR= hazard ratio; CI= confidence interval; BMI = body mass index; LDL= Low-Density Lipoprotein cholesterol.

* Adjusted for baseline covariates (age, sex, country of birth, education levels, marital status, type of occupation, physical activity, smoking status, alcohol drinking, physical activity, BMI, waist circumference, hypertension, cardiovascular diseases, LDL cholesterol, statin prescription).

† Adjusted for diabetes status at enrollment, baseline covariates (age, sex, country of birth, education levels, marital status, type of occupation, physical activity, smoking status, alcohol drinking, physical activity, BMI, waist circumference, hypertension, cardiovascular diseases, LDL cholesterol, statin prescription, elevated depressive symptoms at enrollment), and time-varying covariates (BMI, waist circumference, hypertension, cardiovascular diseases, statin prescription) at the follow-up visit.

**Table 3:** Joint effect estimates (95% CIs) for diabetes and subsequent elevated depressive symptoms on cardiovascular mortality and all-cause mortality using marginal structural models adjusting for baseline and time-varying confounders and right censoring due to loss to follow-up

Outcomes		Cardiovascular mortality		All-cause mortality	
		
Diabetes at enrollment	Elevated depressive symptoms at follow-up	Number of events	Adjusted HR (95% CI) ^*^,^†^	Number of events	Adjusted HR (95% CI) ^*^,^†^
No	No	50/601	Ref	86/601	Ref
Yes	No	43/249	1.82 (1.12 – 3.02)	64/249	2.10 (1.36 – 3.30)
No	Yes	21/179	1.09 (0.46 – 2.17)	38/179	1.13 (0.65 – 1.88)
Yes	Yes	34/107	5.78 (3.02 – 11.97)	43/107	4.32 (2.41 – 7.31)
HR for the interaction term (multiplicative scale)^‡^			2.94 (1.07 – 8.39)		1.80 (0.81 – 4.35)
RERI (additive scale) ^‡^			3.79 (1.05 – 9.81)		2.02 (0.01 – 5.08)

HR, hazard ratio; CI, confidence interval; RERI, relative excess risk due to interaction; BMI, body mass index; LDL, Low-Density Lipoprotein cholesterol.

* Inverse probability of treatment weights was applied to adjust for baseline covariates (age, sex, country of birth, education levels, marital status, type of occupation, physical activity, smoking status, alcohol drinking, physical activity, BMI, waist circumference, hypertension, cardiovascular diseases, LDL cholesterol, statin prescription, elevated depressive symptoms at enrollment) and covariates at the follow-up visit (BMI, waist circumference, hypertension, cardiovascular diseases, statin prescription). Inverse probability of censoring weights was also applied to adjust for right censoring at the follow-up visit due to loss to follow-up.

† 1000 iterations were performed for bootstrapping to estimate 95% CI.

‡ The interaction was significant for cardiovascular mortality on both multiplicative and additive scales, and was significant for all-cause mortality on the additive scale. Multiplicative interaction was calculated by inserting multiplicative term between diabetes and elevated depressive symptoms (HR_DM(yes)_Dep(yes)_ /[HR_DM( *yes* )_D *ep* ( *no* )_ × HR_DM( *no* )___D *ep* ( *yes* )_]; null value = 1), and additive interaction was calculated by RERI (HR_DM(yes)_Dep(yes)_ - HR_DM(yes)_Dep(no)_ - HR_DM(no)_Dep(yes)_ +1; null value = 0).
