# Supplementary material for: Estimating the joint effect of diabetes and subsequent depressive symptoms on mortality among older latinos
Source: Ann Epidemiol. Author manuscript; Available in PMC 2024 Jul 9. (PMC11232658; doi:10.1016/j.annepidem.2021.09.007)
Supplement: Supplementary materials [file NIHMS1858596-supplement-Supplementary_materials.pdf]

-Online Supporting Information-

Supplementary Text

Creation of inverse-probability-treatment weights and inverse-probability-of-censoring weights for a marginal structural model under investigation

Supplementary Figures

1. Distribution of the Center for Epidemiological Studies-Depression (CESD) scores at first follow-up visit according to diabetes status at enrollment

Supplementary Tables

1. Distribution of elevated depressive symptoms at enrollment and the first follow-up visit according to diabetes status at enrollment.

2. Joint effect estimates (95% CIs) for diabetes and elevated depressive symptoms at enrollment with cardiovascular mortality and all-cause mortality using Cox proportional hazard models.

3. Joint effect estimates (95% CIs) for diabetes at enrollment and subsequent depressive symptoms at the first follow-up visit with cardiovascular mortality and all-cause mortality restricting to participants without depressive symptoms at enrollment.

Supplementary Text. Creation of inverse-probability-treatment weights and inverse-probability-of-censoring weights for a marginal structural model under investigation

- Inverse-probability-of-treatment weights (IPTW) were calculated as follows:

$$\frac{pr[DM_1]}{pr[DM_1|\ Cov_1]} \times \frac{pr[Dep_2]}{pr[Dep_2|\ DM_1,Cov_1,Cov_2]}$$

  where pr[.] is the probability function, DM_1 = diabetes at enrollment, Cov_1 = covariates at enrollment, Dep_2 = elevated depressive symptoms at the first follow-up visit, and Cov_2 = covariates at the first follow-up visit. Covariates at enrollment include age, sex, country of birth, education levels, marital status, type of occupation, physical activity, smoking status, alcohol drinking, physical activity, BMI, waist circumference, hypertension, cardiovascular diseases, LDL cholesterol, statin prescription, and elevated depressive symptoms. Covariates at the first follow-up visit (i.e., time-varying covariates) include BMI, waist circumference, hypertension, cardiovascular diseases, and statin prescription.

- Inverse-probability-of-censoring weights (IPCW) were calculated as follows:

$$\frac{pr[C_2{=}0]}{pr[C_2{=}0|\ DM_1,Cov_1]}$$

  where C_2 = censoring (1, censored; 0, not censored) at the first follow-up visit, DM_1 = diabetes at enrollment, and Cov_1 = covariates at enrollment. Covariates at enrollment include age, sex, country of birth, education levels, marital status, type of occupation, physical activity, smoking status, alcohol drinking, physical activity, BMI, waist circumference, hypertension, cardiovascular diseases, LDL cholesterol, statin prescription, and elevated depressive symptoms.

- The final weights for each participant were created by multiplying the IPTW and the IPCW:

$$\frac{pr[DM_1]}{pr[DM_1|\ Cov_1]} \times \frac{pr[Dep_2]}{pr[Dep_2|\ DM_1,Cov_1,Cov_2]} \times \frac{pr[C_2{=}0]}{pr[C_2{=}0|\ DM_1,Cov_1]}$$

Supplementary Figure 1. Distribution of the Center for Epidemiological Studies-Depression (CESD) scores at first follow-up visit according to diabetes status at enrollment

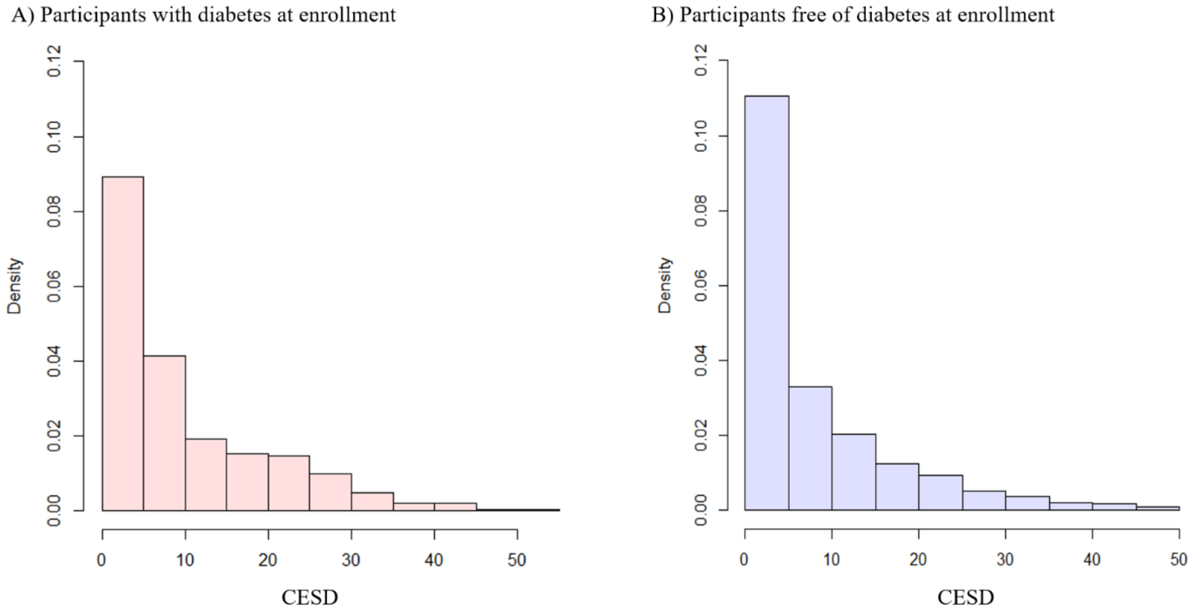

The percentage of reporting anti-depressant use was 11.3% and 6.9% among participants with diabetes at enrollment and those free of diabetes at enrollment, respectively.

Supplementary Table 1. Distribution of elevated depressive symptoms at enrollment and the first follow-up visit according to diabetes status at enrollment.

**A) Total population**		Elevated depressive symptoms at follow-up		
		Yes	No	Total
Elevated depressive symptoms at enrollment	Yes	177	148	325
	No	109	702	811
	Total	286	850	1136
**B) Participants with diabetes at enrollment**		Elevated depressive symptoms at follow-up		
		Yes	No	Total
Elevated depressive symptoms at enrollment	Yes	66	53	119
	No	41	196	237
	Total	107	249	356
**C) Participants free of diabetes at enrollment**		Elevated depressive symptoms at follow-up		
		Yes	No	Total
Elevated depressive symptoms at enrollment	Yes	111	95	206
	No	68	506	574
	Total	179	601	780

Supplementary Table 2. Joint effect estimates (95% CIs) for diabetes and elevated depressive symptoms at enrollment with cardiovascular mortality and all-cause mortality using Cox proportional hazard models.

**Outcomes**		**Cardiovascular mortality**		**All-cause mortality**	
**Diabetes at enrollment**	**Elevated depressive symptoms at enrollment**	**Number of Events**	**Adjusted HR (95% CI)** [a, b]	**Number of Events**	**Adjusted HR (95% CI)** [a b]
No	No	62/724	Ref	111/724	Ref
Yes	No	78/318	2.56 (1.75 to 3.65)	106/318	2.12 (1.59 to 2.87)
No	Yes	40/283	1.38 (0.86 to 2.18)	67/283	1.29 (0.94 to 1.79)
Yes	Yes	38/170	2.22 (1.39 to 2.18)	57/170	2.12 (1.49 to 3.16)
HR for the interaction term (multiplicative scale)			0.64 (0.35 to 1.18)		0.78 (0.48 to 1.27)
RERI (additive scale)			-0.68 (-2.00 to 0.48)		-0.26 (-1.23 to 0.59)

HR, hazard ratio; CI, confidence interval; RERI, relative excess risk due to interaction; BMI, body mass index; LDL, Low-Density Lipoprotein cholesterol.

[a]Adjusted for baseline covariates (age, sex, country of birth, education levels, marital status, type of occupation, physical activity, smoking status, alcohol drinking, physical activity, BMI, waist circumference, hypertension, cardiovascular diseases, LDL cholesterol, statin prescription).

[b] 1000 iterations were performed for bootstrapping to estimate 95% CI.

Supplementary Table 3. Joint effect estimates (95% CIs) for diabetes at enrollment and subsequent depressive symptoms at the first follow-up visit with cardiovascular mortality and all-cause mortality restricting participants without depressive symptoms at enrollment.

**Outcomes**		**Cardiovascular mortality**		**All-cause mortality**	
**Diabetes at enrollment**	**Elevated depressive symptoms at follow-up**	**Number of Events**	**Adjusted HR (95% CI)** [a]	**Number of Events**	**Adjusted HR (95% CI)** [a]
No	No	38/506	Ref	68/506	Ref
Yes	No	38/196	2.40 (1.34 to 4.35)	53/196	2.19 (1.47 to 3.48)
No	Yes	6/68	0.75 (0.12 to 2.11)	12/68	0.93 (0.33 to 1.98)
Yes	Yes	15/41	7.12 (2.86 to 18.06)	19/41	5.23 (2.56 to 10.51)
HR for the interaction term (multiplicative scale)			4.28 (0.96 to 28.90)		2.58 (0.92 to 8.66)
RERI (additive scale)			4.93 (-0.48 to 14.75)		2.98 (0.17 to 8.43)

HR, hazard ratio; CI, confidence interval; RERI, relative excess risk due to interaction; BMI, body mass index; LDL, Low-Density Lipoprotein cholesterol.

[a] Inverse probability of treatment weights was applied to adjust for baseline covariates (age, sex, country of birth, education levels, marital status, type of occupation, physical activity, smoking status, alcohol drinking, physical activity, BMI, waist circumference, hypertension, cardiovascular diseases, LDL cholesterol, statin prescription) and covariates at the first follow-up visit (BMI, waist circumference, hypertension, cardiovascular diseases, statin prescription). Inverse probability of censoring weights was also applied to adjust for right censoring at the first follow-up visit due to loss to follow-up.

[b] 1000 iterations were performed for bootstrapping to estimate 95% CI.
